# Hypothesis: Emerging Roles for Aryl Hydrocarbon Receptor in Orchestrating CoV-2-Related Inflammation †

**DOI:** 10.3390/cells11040648

**Published:** 2022-02-13

**Authors:** Tiziana Guarnieri

**Affiliations:** 1 Cell Physiology Laboratory, Department of Biological, Geological and Environmental Sciences (BiGeA), Alma Mater Studiorum Università di Bologna, 40126 Bologna, Italy; tiziana.guarnieri@unibo.it; 2 Interuniversity Consortium “Istituto Nazionale Biostrutture e Biosistemi” (INBB–Biostructures and Biosystems National Institute), 00136 Rome, Italy; 3 Interdepartmental Center for Industrial Research in Life Sciences and Technologies, University of Bologna, 40126 Bologna, Italy

**Keywords:** aryl hydrocarbon receptor, inflammation, SARS-CoV-2, COVID-19, kynurenine

## Abstract

Severe Acute Respiratory Syndrome Coronavirus-2 (SARS-CoV-2) is the pathogenic agent of Coronavirus-Induced Disease-2019 (COVID-19), a multi-organ syndrome which primarily targets the respiratory system. In this review, considering the large amount of data pointing out the role of the Aryl hydrocarbon Receptor (AhR) in the inflammatory response and in the modulation of innate and adaptive immunity, we describe some mechanisms that strongly suggest its involvement in the management of COVID-19′s inflammatory framework. It regulates both the expression of Angiotensin Converting Enzyme-2 (ACE-2) and its stabilizing partner, the Broad neutral Amino acid Transporter 1 (B^0^AT1). It induces Indolamine 2,3 dioxygenase (IDO-1), the enzyme which, starting from Tryptophan (Trp), produces Kynurenine (Kyn, Beta-Anthraniloyl-L-Alanine). The accumulation of Kyn and the depletion of Trp arrest T cell growth and induce apoptosis, setting up an immune-tolerant condition, whereas AhR and interferon type I (IFN-I) build a mutual inhibitory loop that also involves NF-kB and limits the innate response. AhR/Kyn binding boosts the production of Interleukin-6 (IL-6), thus reinforcing the inflammatory state and counteracting the IDO-dependent immune tolerance in the later stage of COVID-19. Taken together, these data depict a framework where sufficient clues suggest the possible participation of AhR in the management of COVID-19 inflammation, thus indicating an additional therapeutic target for this disease.

## 1. COVID-19’s Etiology and Symptoms: A Short Description

According to the World Health Organization (WHO), lower tract respiratory diseases ranked fourth in the list of the top ten global causes of death in the period from 2000 through the end of 2019 [1]. It is likely that the current COVID-19 pandemic will influence the position of these communicable pathologies in future rankings. At the time that this article was submitted for peer review, the world number of deaths due to this devastating pandemic exceeded 5.1 million out of a total of approximately 254 million confirmed cases between February 2019 and November 2021 [2]. 

The etiology of COVID-19 has been identified in a positive-sense, single-stranded RNA coronavirus 2 (CoV-2), which drives an infection that primarily targets the lower respiratory tract, resulting in the Severe Acute Respiratory Syndrome (SARS). Symptoms vary from mild to severe intensity and include evident respiratory distress, resulting in respiratory insufficiency and so-called “air hunger” [3]. According to the Centers for Disease Control and Prevention (CDC), a dry and persistent cough, fever and headache are symptoms that most frequently occur in the initial phase. They can be associated with tachycardia and fatigue, which are consequences of respiratory insufficiency; the loss of sense of taste and smell, which is a signal of neurological involvement; and skin rashes and color changes in fingers and toes, evidencing the engagement of the immune system. Another common target of SARS-CoV-2 is the digestive system, the imbalance of which is shown by diarrhea and nausea [4]. Blood clotting and thrombosis, probably due to immunothrombotic dysregulation, occur in the later stages of COVID-19 and are the most worrisome complications occurring in about 30% of hospitalized cases [5,6]. 

## 2. ACE-2, the Highest Common Denominator of COVID-19 Targets

The common factor of this multiplicity of cytologic targets of SARS-CoV-2 is the presence in the cell membrane of Angiotensin (Ang)-Converting Enzyme 2 (ACE-2), a homologue of the more widely known ACE [7]. ACE-2 is expressed in different human cell types, among which are airway cells [8] enterocytes [9], macrophages [10], endothelial cells [11], brain cells [12], testis cells and kidney cells [13]. ACE-2 is an integral membrane protein, with the interesting exception of the brain, where it is present in the cytoplasm of neuronal cell bodies [12]. Until a few years ago, ACE-2 was known mostly for its ability to counterbalance the vasoconstrictive effects of Ang II, which is produced through the catalytic action of ACE. ACE-2 catalyzes the transformation of Ang II into Angiotensin 1–7 [(Ang-(1–7)], which has a vasodilatory effect. A decade ago, some authors also described its antifibrotic and antiproliferative effects [14,15]. This protein is pleiotropic: it has enzymatic activity through which it hydrolyzes a single hydrophobic/basic residue from the -COOH terminus of its substrates. It is also a chaperone of the neutral amino acid transporter B^0^AT1 in the small intestine, where it is highly expressed [16]. At present, the most infamous function of the membrane form of ACE-2 is its ability to bind the viral subunit 1 of the Spike proteins (S1) that are exposed in a crown-like (corona) shape on the external surface of SARS-CoV-2. Thus, the viral-binding capacity of ACE-2 is the keystone in COVID-19 infection [17], as it allows for virus entry into target cells, where it replicates, thus giving rise to a plethora of symptoms, the nature and intensity of which are the subjects of intense study. In general, the level of membrane ACE-2 expression and the viral load are proportional to the intensity of symptoms and the severity of disease, but this equation is not always true, as it intersects with the so-called host factors, which include age and phenotypic sex, bodily composition, clinical history and the presence of autoimmune, inflammatory, and coagulation disorders [18]. Assuming that most of the symptoms are a consequence of an infection-driven inflammatory process, its amplification and/or its sequelae, it is mandatory to identify the connection between ACE-2 activation and the inflammatory onset, propagation and amplification. Certainly, ACE-2 expression is greatly increased from inflammation, as documented in inflamed enterocytes [19,20], pneumocytes [21] and airway cells, where the *ACE-2* gene has been described as a human interferon-stimulated gene (ISG) [22].

## 3. AhR Modulates ACE-2 and B^0^AT1

In a recent correspondence paper, Jiadi Lv and coworkers [23] reported that the Aryl hydrocarbon Receptor (AhR), the transcription factor mostly known as a sensor for xenobiotics, regulates the expression of *ACE-2* in SARS-CoV-2-infected primates. Here, they observed that AhR binds the promoter of *ACE-2*, thus increasing its expression. Consequently, this favors a higher viral load and a greater severity of histopathological damage. Considering that AhR is a ligand-activated transcription factor, they investigated the effect of some well-known AhR activators and inhibitors. In BEAS-2B, a non-tumorigenic human lung epithelial cell line and in mice, they observed that Kyn, a well-known endogenous agonist of AhR, stimulates ACE-2 gene transcription and protein expression. They obtained similar results using FICZ (6-formylindolo (3,2b) carbazole), an endogenous agonist of AhR, derived from the photooxidation of Trp. Given these data, they hypothesized that AhR regulates ACE-2 expression. Then, they tested this possible link in SARS-CoV-2 infection. After Kyn pretreatment in BEAS-2B infected with SARS-CoV-2, they verified a higher viral load, coupled with a greater *ACE-2* expression. Consistently, in the lung tissue of SARS-CoV-2-infected macaques, Lv’s group observed the increase in AhR activation. After the administration of the AhR inhibitor CH223191, the decrease in *ACE-2* expression and viral load clearly confirmed that ACE-2 expression can be modulated by AhR. This simple yet elegant study highlights an interesting clue which links the expression of ACE-2 to an AhR-mediated modulation. In the small intestine, ACE-2 stabilization is achieved by the association with Broad neutral Amino acid Transporter 1 (B^0^AT1) (encoded by the *SLC6A19 (Solute Carrier Family 6 Member 19* gene). B^0^AT1, which is chaperoned by ACE-2 and mediates the entry of neutral amino acids into intestinal, kidney and liver cells [24]. At the beginning of 2020, Yan and coworkers proposed the binding between the trimeric form of the SARS-CoV-2 protein Spike and ACE-2. Using cryo-electron microscopy techniques, they also showed that ACE-2 forms a functional unit with B^0^AT1 and provided evidence that in this complex, ACE-2 is present as a dimer. Thus, they hypothesized the binding of two trimers of S protein to the ACE-2 dimer present in the ACE-2:B^0^AT1] complex. Already in 2018, Tian group had demonstrated that in HepG2 cells treated by dioxin and dioxin-like molecules, AhR targets the *SCL6A19* gene [25]. Considering these data showing the AhR modulation of ACE-2 and B^0^AT1 in cell types expressing both proteins, it is tempting to hypothesize that in SARS-CoV-2-infected cells, activated AhR could influence the interaction between the S protein and the ACE-2:B^0^AT1 complex. This mechanism seems to be plausible in the gut, which presents ACE-2:B^0^AT1 dimers and could explain the intestinal symptoms of COVID-19, which are frequently precocious in COVID-19 [4,25]. Conversely, as already described, lung cells do not express B^0^AT1, and the entry of the virus is conditioned by the presence of TMPRSS2, a transmembrane serine protease that is necessary for the cleavage of S protein that precedes the priming of ACE-2, the fusion of membranes and the cytosolic release of viral RNA [26]. Viral entry is also mediated by cathepsin B and L (CTS B/L), two endosomal cysteine proteases participating in the cleavage of the S protein. In particular, in COVID-19-affected patients, elevated levels of serum CTS L/B are reported [27,28]. In a recent paper by Muus and coworkers, SARS-CoV-2 infection susceptibility was shown to be associated with increased levels of ACE-2, Transmembrane Protease Serine 2 (TMPRSS2) and Cathepsin L (CTSL). In COVID-19-infected subjects, nasal, lung and gut epithelial cells express *ACE-2* and *TMPRSS2* and a genic panel characterized by the activation of the proinflammatory gene *IL-6*, its receptor *IL-6R*, its co-receptor *IL-6ST*, Interleukin1 receptor (*IL-1R*), Tumor Necrosis Factor (*TNF*)-related pathways and complement genes. The authors hypothesize that SARS-CoV-2 infection may stimulate the expression of this proinflammatory genic panel, which is probably involved in mediating some aspects of immune functions and the interaction between epithelial cells and macrophages [29]. This association is not a surprise, as we know that these molecules participate in the onset and development of inflammation, which is the feeding substrate of most viral infections. *TMPRSS2* is an Androgen Receptor (AR) target gene, whose expression is upregulated by male sexual hormones testosterone (T) and dihydrotestosterone (DHT) in human prostate cells. Here, Arabnezhab and colleagues [30] demonstrated that, once activated by the endogenous agonist FICZ, AhR interferes in the AR-mediated transcription of some genes, including *TMPRSS2.* The dependency of TMPRSS2 expression on male sexual steroids could be a contributing factor in explaining the higher incidence of COVID-19 in males. However, it must be considered that these data are preliminary and that the anti-androgenic effect of FICZ, which also reverberates on DHT and Prostate-Specific Antigen (PSA) levels, could be a serious side-effect of a hypothetical FICZ-based anti-COVID-19 therapy. An interesting discrepancy concerning AhR involvement in the management of SARS-CoV-2 infection comes from Tanimoto’s study [31] that recently highlighted an inverse correlation between *ACE-2* expression and AHR activation in non-respiratory mammalian cell lines using its agonists FICZ, omeprazole (OMP) and Cigarette Smoke Extract (CSE). According to Tanimoto’s data, the more the AHR pathway is activated, the less *ACE-2* is expressed, and consequently, the viral load decreases. However, Tanimoto’s group used hepatic HepG2 and renal Vero E6 cells. The higher inhibition of *ACE-2* is observed in HepG2 using CSE. CSE contains a mixture of agonists and antagonists plus many unknown molecules, which probably affect not only *AhR* but also *ACE-2* expression. In addition, they also observed an inverse correlation between *CYP1A1* induction and *ACE-2* expression. This suggests that AhR’s role in the liver could still be related to detoxification rather than participation in inflammatory processes. In our opinion, these data should be integrated with those obtained in cells from the respiratory system. SARS-CoV-2 primarily affects the respiratory system, where the expression of ACE-2 is directly proportional to the expression and activation of AhR, as suggested by the studies of Lv [23]. Here, SARS-CoV-2 can also gain access through dipeptidyl peptidase-4 (DPP4) that is inhibited from activated AhR, which could explain the apparent protection of smokers from COVID-19. In liver and kidney, which are the source of the cell lines used by Tanimoto, *ACE-2* expression probably has a more complex regulation. Tanimoto proposes that the inhibition of *ACE-2* expression could be driven by the functional interaction between AhR and the transcription factors p300 and JUN that bind to the *ACE-2* gene. In addition, he hypothesized that in HepG2, treatment with some AhR agonists interferes with the dialogue between GATA4 and AhR, which could be the origin of the downregulation of *ACE-2* expression.

## 4. AhR Signaling

**Figure 1:**
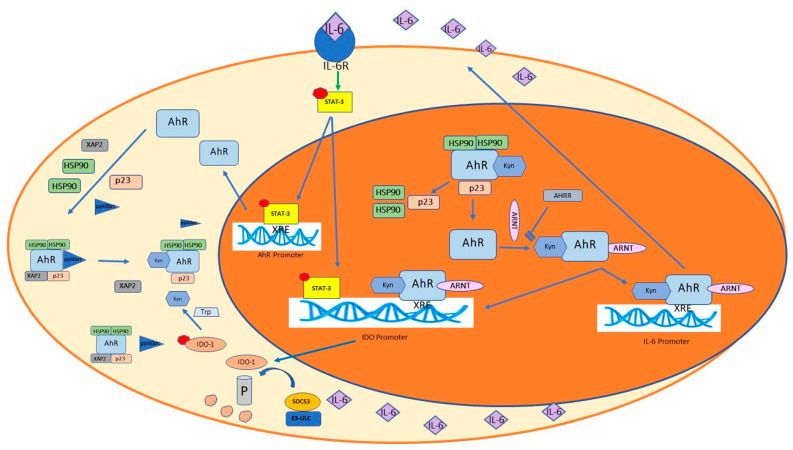
Bi-modal activation of AhR. Kyn activates AhR, which in an inflammatory context, binds to its response element in the promoter of *IL-6*, thus participating in IL-6′s endogenous production and the amplification of the inflammatory state. IL-6 binding to its receptor IL-6R results in STAT-3 activation, which binds to IDO-1 and AhR promoters, thus endowing *IDO-1* and *AhR* gene expression. In the cytoplasm, IDO-1 catalyzes the formation of Kyn from Trp; Kyn binds and activates AhR, which similarly to STAT-3, binds to the IDO-1 promoter and activates *IDO-1* expression. IDO-1 enzymatic activation is also obtained by pp60src, which detaches from the AhR inactivating complex and phosphorylates IDO-1, thus completing this inflammatory loop. High levels of IL-6 favor the upregulation of SOCS3, which engages the E3 ubiquitin ligase complex (E3-ULC), thus driving cytoplasmic IDO-1 to proteasomal degradation. In parallel, STAT-3 sustains the expression of AhR, which in turn, stimulates the expression of IL6, thus setting a self-sustaining autoinflammatory loop.

AhR, a widely expressed transcription factor, is a basic helix-loop-helix (bHLH)/PAS protein, but differently from other (bHLH)/PAS proteins, it is not embedded in membranes and is located in the cytoplasm. Here, it is inactivated in a multi molecular complex composed of: 1. two 90-kDa Heat Shock Proteins (HSP90s) that stabilize AhR in conformation with a high affinity for its ligands [32]; 2. one Immunophilin-like molecule, known as AhR-Interacting Protein (AIP, also called XAP2 or Ara9), which stabilizes the interaction between AhR and HSP90; 3. one p23 protein, which protects AhR from ubiquitination and degradation [33]; and 4. the signaling partner protein tyrosine kinase pp60src, which is released after ligand binding and phosphorylates different targets (Figure 1). In inflammation, for example, pp60src interacts with the Epithelial Growth Factor Receptor (EGFR), thus activating the cascade of Mitogen-Activated Protein Kinase (MAPK). This stimulates the transcription of Cyclooxygenase-2 (*COX-2*) and Matrix Metalloproteinase-1 (*MMP-1*) genes. [34]. After ligand binding, AIP is released from the complex, which changes its conformation and unmasks a basic amino acid segment (from 19 to 39), known as the Nuclear Localization Signal (NLS) [35,36]. Activated AhR can fuel both genomic and non-genomic pathways [37,38]. In genomic pathways, due to nuclear importing components, AhR shuttles into the nucleus, releases the remaining inactivating molecules and binds to AhR nuclear translocator/hypoxia-inducible factor-1β (ARNT/HIF-1β). The AhR/ARNT heterodimer binds to cognate DNA motifs in the XRE (or DRE) of target genes. Reporting the classification of Bock [39], target genes are involved in AhR repression (*AHRR*), antioxidative functions (*NRF2*), chemical defense (phase I enzymes *CYP1A1, CYP1A2, CYP1B1*; phase II enzymes *UGT1, GSTA1/2, ABCG2*), microbial defense (*NADPH oxidase/P40^phox^*), organ development (*P21CIP, P27KIP, P40PHOX, BLIMP1*), immunity and inflammation (*C-KIT, IL-1β, IL-6, IL-17, IL-22, CXCL5, IDO/TDO*), reproduction (*CYP 19*) and energy homeostasis (*TiPARP, CD36, CD38*). Thus, from this list, we can argue that AhR functions are not limited to biosensor functions, which, in effect, were acquired more recently. 

It is generally assumed that AhR expanded its functions during eukaryotic cell evolution. Originally involved in the control of the cell cycle, differentiation and proliferation, this molecule gradually added to these functions the sensing of environmental molecules, especially those containing Aromatic Hydrocarbons. During the evolution of our planet, combustion processes gradually accumulated in the atmosphere Halogenated Aromatic Hydrocarbons (HAH), Polycyclic Aromatic Hydrocarbons (PAH) and a plethora of molecules resulting from the oxidation of organic and inorganic matter. Due to molecular evolution and the presence of multiple sites able to bind these xenobiotics, AhR added detoxification properties to its original functions. AhR is able to bind not only exogenous molecules, but also endogenous ligands from alimentary sources or the tryptophan catabolism of microbiota [40]. In mammals, it participates in nervous, cardiovascular, and digestive functions and is a pivotal element in the regulation of the immune system and circadian rhythms [41]. Its dysregulation can have a critical role in some important diseases. A wealth of data has described its participation in neoplastic onset and progression [42] in the breast [43], colon [44], head and neck [45], pancreas [46], liver [47], and skin [48]. The number of inflammation-related genes targeted from AhR underlines its involvement in inflammation [38,39]. 

## 5. AhR Is Activated in the Viral Inflammatory Environment

Typically, the cell entry of viruses is followed by replication, which is recognized from Pattern Recognition Receptors (PRRs), a viral replication detection system. After activation, they oligomerize and engage transcription factors, among which are Interferon Regulatory Factors (IRFs) and the Nuclear Factor kappa-light-chain-enhancer of activated B cells (NF-kB). Consequently, innate immune response, mediated by IFN-I and IFN-III and their effectors, are set. [49]. The biochemical and immunological features of COVID-19-related inflammation, in comparison with other respiratory viruses, were extensively described by Daniel Blanco-Melo and coworkers in a recent review [50]. Here, they underlined that, in contrast with common viral infections, the host’s transcriptional response to SARS-CoV-2 entry is characterized by a low production of IFN-I and IFN-III and, consequently, from a reduced ISG response. In addition to this insufficient innate antiviral response, there is also an extremely strong inflammatory response (the so-called “inflammatory storm”) sustained by high levels of chemokines, such as interleukin-8 (CXCL-8), monocyte-attracting protein-1 (MCP-1/CCL-2) and several cytokines. In particular, IFNγ—a cytokine that is produced during inflammation to counteract viral infections and to exert an immunomodulatory influence—and other inflammatory mediators, such as Il-1β, IL-6 and TNFα [51,52], are produced and upregulate indoleamine 2,3-dioxygenase (IDO-1), the rate-limiting enzyme in the catabolism of Trp. 

The upregulation of IDO-1 has been described in other viral infections, such as hepatitis C infection. Here, IDO-1 endows T cells apoptosis by depriving them of the essential amino acid Trp, which is transformed in Kyn [53]. Kyn is also, produced in the liver by the enzyme tryptophan dioxygenase (TDO) and during the immune response in many bodily districtsby the enzyme indolamine 2,3-dioxygenase 1/2 (IDO-1/IDO-2). The decrease in the Trp/Kyn ratio is a marker of IDO/TDO activity. Kyn is an endogenous activating ligand of AhR that regulates the expression of the gene encoding for B^0^AT1, the neutral amino acids transporter that mediates the Trp influx/Kyn efflux and stabilizes ACE-2 [25].

**Figure 2:**
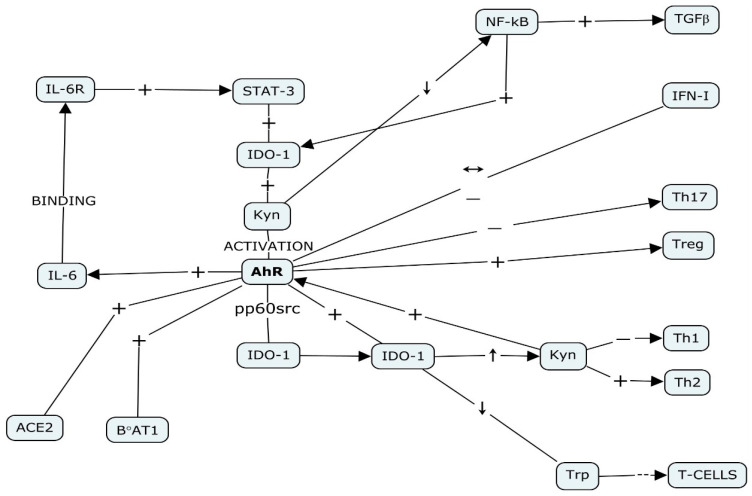
The AhR management of inflammation in COVID-19. Concept map summarizing AhR’s role in the inflammatory response and in the modulation of innate and adaptive immunity. The right side of the diagram summarizes the effects of AhR on innate (TiPARP-mediated inhibition of IFN-I) and acquired immunity (shift in Treg/Th17 balance). The left side of the diagram summarizes the IL-6_STAT-3_IDO_Kyn_AhR-driven auto inflammatory loop and AhR’s influence on ACE-2 and B^0^AT1 expression. The map was drawn using the freeware software CmapTools, developed by the Florida Institute for Human and Machine Cognition (IHMC) (Cmap | CmapTools (ihmc.us), accessed on 10 February 2022).

In the viral inflammatory framework, as extensively described by Torti and coworkers [54], the Kyn-mediated activation of AhR is a possible significant part of the anti-viral strategy of host cells that we also hypothesize in COVID-19 infection. In addition, in inflammatory contexts where there are high levels of IL-6, it was already proposed [43,55] that IL-6 induces *IDO-1* expression via the Signal Transducer and Activator of Transcription-3 (STAT-3) activation. In the cytoplasm, IDO-1 catalyzes the transformation of Trp in Kyn, which in turn, activates AhR. AhR binds to its response element XRE (or DRE) in the promoter of *IL-6*, thus sustaining IL-6 endogenous production and the amplification of the inflammatory state (Figure 1 and Figure 2). As we will consider later, the increase in Kyn levels has an immune-depressive effect, since it leads to the AhR-mediated differentiation of Treg cells [56]. As recently reviewed by Pallotta and colleagues [57], in Dendritic Cells (DCs), IDO-1 is able to modulate immune functions in a context-dependent modality. In an inflammatory context driven by IFNγ, IDO-1 is activated and drives the production of Kyn. Kyn binding activates AhR and upregulates the expression of *IDO-1*; in IL-6-driven inflammation, IDO-1 is ubiquitinated and degraded by means of SOCS3. This counteracts the IDO-dependent tolerogenesis and favors the establishment of an inflammatory environment. When TGF-β concentration is high, IDO-1 is involved in the activation of the “non-canonical” anti-inflammatory isoform of NF-κB. In these conditions, NF-κB induces the expression of *IDO-1* and *TGF-β* genes, thus favoring the onset of a stable immune-tolerant environment [58]. Considering that the less the immune response is efficient, the more infections are successful, it is tempting to wonder whether the balance between these mechanisms could explain, at least in part, the different levels of severity in SARS-CoV-2-induced inflammation. We know that its intensity and duration vary depending on the intensity and duration of the triggering stimulus and the effectiveness of the immune response, which in this case, includes the clearing of the virus. In the case of SARS-CoV-2, the acute inflammation quickly turns in an “inflammatory storm”, mainly sustained by the secretion of inflammatory cytokines, among which IL-6 is the most prominent [59]. As early as 2014, Stobbe-Maicherski’s group showed that oncostatin M (OSM), a cytokine belonging to the (IL)-6-type family, is able to induce *AhR* expression through a direct interaction of its downstream transcription factor STAT3 and a STAT-binding motif in the *AhR* promoter [60]. Recently, we obtained similar results in a non-tumorigenic breast cell line (not published). Consequently, it is tempting to hypothesize that, in SARS-CoV-2-mediated infection, the initial involvement of AhR could be mainly evoked by increased levels of its agonist Kyn. This phase is short-lived and coincides with the setting up of an immune-tolerant environment. Then, the dramatic increase in IL-6 levels during the “cytokine storm” stimulates a significant SOCS-3-mediated inactivation of IDO-1, a rapid decline in Kyn and a decrease in the immunotolerant condition [58]. In this hyper-inflammatory environment, we hypothesize that *AhR* expression could be mainly stimulated by STAT3, which sustains both *AhR* and *IDO-1* transcription (Figure 1). As already shown by some authors [61,62], in inflammatory environments, AhR itself stimulates *IL-6* expression, thus participating in the maintenance of its high levels and originating an auto-inflammatory loop. Regardless, due to the unselective AhR binding capacity, we cannot exclude its participation in different COVID-19-related inflammatory settings.

*ACE-2* expression is stimulated in inflammatory conditions. Consistently, in an interesting paper published in May 2020, Chen and colleagues [63] described a retrospective analysis of 21 patients whose COVID-19 symptoms varied from mild to severe. Although the number of cases reported is limited, emerging data are consistent with a general picture where males are more affected than women and there is a pattern of immune failure coupled with hyperinflammation. In particular, immune issues are highlighted by lymphopenia due to a decrease in CD4^+^ (helper) and CD8^+^ (cytotoxic) T cells. A dismal inflammatory state is evidenced by hypoalbuminemia and high serum levels of some inflammatory cytokines, including IL-6, IL10, IL2 and TNFα. In this regard, an attracting parallelism is the immune dysregulation that follows TCDD-mediated AhR activation in CD4^+^ and CD8^+^ T-cell lymphocytes, where the cytotoxic response is abolished [64]. This suggests that AhR participation in COVID-19 disease could be evocated and impact not only inflammation development, but also the immune response. As we will see, this hypothesis was confirmed.

## 6. AhR Participates in COVID-19’s Immune-Inflammatory Imbalance

The comprehension of AhR’s role in the COVID-19 framework must consider its participation in human physiology and, in particular, in inflammatory and immune processes [65,66], where AhR is involved in the regulation of both innate and adaptive immunity, as it influences both DCs and T lymphocytes. In DCs, it decreases the expression of the Major Histocompatibility Complex II (MHC II). It also regulates the production of inflammatory cytokines, such as IL-6, IL12, IL15, and IL18, which are usually produced during DC differentiation [67]. DCs differentiation takes place upon their exposure to T cells, viral/bacterial components or proinflammatory molecules, such as granulocyte-macrophage colony-stimulating factor (GM-CSF), IFNα, and inflammatory cytokines such as IL-6 and TNFα. This point is of pivotal importance, due to the crosstalk between several inflammatory molecules such as IL-6, TNFα and the AhR pathway [60,61]. This interplay also reverberates on the differentiation of Th17 and Treg_,_ in particular in type 1 regulatory T cell (T_R_1) stabilization. AhR can deeply affect T cells metabolism, which can be modulated depending on the AhR ligand. It has been demonstrated that the differentiation of T_R_1 cells is the result of the sequential collaboration of Hypoxia Inducible Factor-1α (HIF-1α) and AhR. In physiologic settings, AhR endows the degradation of HIF-1α, while in inflammation, which is frequently associated to hypoxia, HIF-1α inactivates AHR, thus interfering with T_R_1 cell differentiation [68]. AhR is also abundantly expressed in DCs [69]. Recently, Cheong and Sun described the switch of DCs toward an immunotolerant phenotype consequent to the activation of (IDO-1) and tryptophan 2,3-dioxygenase 2 (TDO2) in the tumor microenvironment [70]. These enzymes catalyze the transformation of Trp in Kyn. Consequently, there is a constant subtraction of Trp and the accumulation of Trp tRNA. This stimulates the stress response kinase General Control Nonderepressible 2 (GCN2) to halt T cells activation. In parallel, the increase in Kyn activates AhR, which endows the transcription of mediators of immune suppression, the development of regulatory T cell populations and the production of inflammatory mediators, including CCL2 and IL6. (Figure 2). This mechanism is proposed as an explanation of tumor immune escape but, recently, it has also been considered in SARS-CoV-2-induced inflammation. As demonstrated by Liu [52], in SARS-CoV-2-infected pulmonary cells and in mice, the IDO–Kyn–AHR metabolic circuitry is activated from IFN-β and IFN-γ. IFNs are produced in the first phase of viral invasion by immune cells. In this case, their anti-viral strategy is based on IFN-β and IFN-γ production, which engage AhR through the IDO-1-dependent production of Kyn. In particular, Kyn-activated AhR binds to the promoter of several genes that encode for mucins. Mucins are glycoproteins produced by respiratory epithelial tissue, the production of which is greatly increased in COVID-19 pathology, as ascertained in the bronchoalveolar lavage fluid (BALF) of COVID-19 patients and in post-mortem examinations. The result is an increased production of mucus, probably to hinder viral invasion in the preliminary phases of COVID-19 infection. This possibility is also suggested by the fact that in antigen-presenting cells, IDO-1, and thus Kyn, is also induced by some proinflammatory molecules, including IL-1, IL-6, TNF-α, and LPS and CpG oligonucleotides via Type I Interferons (IFN-Is) [71,72,73,74].

### 6.1. Interferons

Interferons are a group of proteins that, as suggested by their name, interfere with the replication and spreading of viruses. They stimulate the transcription of a myriad of ISGs, whose products are an extremely diversified panel of molecules. Some of them actively participate in the antiviral response, in the setting of a long-lasting adaptive immunity and the control of proliferation in repaired tissues. Early stages of innate immune response against viral infections are mediated by Type I IFNs, which in COVID-19, are referred to as “weak” and transitory [75]. Among IFNs, IFN-Is are a large family of structurally related cytokines including IFN-α (13 different subtypes), IFN-β, IFN-ε, IFN-κ and IFN-ω. They are produced mainly by plasmacytoid Dendritic Cells (pDCs) when Pathogen-Associated Molecular Patterns (PAMPs) associated with viral nucleic acids are detected by cytoplasmic sensors belonging to the family of Retinoic acid-Inducible Gene I (RIG-1)-like receptors (RLSs) and Melanoma Differentiation-Associated gene 5 (MDA-5). This interaction engenders a complex pathway that culminates in the production and secretion of IFN-I family molecules. In SARS-CoV-2 infection, secreted IFN-Is—mainly IFNα and IFNβ—bind interferon alpha and beta receptors (IFNARα and IFNARβ) in target cells, including type II pneumocytes, cardiomyocytes, endothelial cells, enterocytes, hepatocytes, and astrocytes [29]. IFNAR binding originates an intracellular pathway where phosphorylated STAT1 and STAT2 heterodimerize and associate with a DNA-binding protein called IFN regulatory factor 9 (IRF9) [74,75]. This complex, named IFN-stimulated growth factor 3 (ISGF3), moves to the nucleus and targets interferon-stimulated response elements (ISREs) in the promoters of ISG. [76]. *ACE-2* has been described as an ISG in an in vitro model of human airway cells [22]. ISGs also include antiviral effectors and positive/negative IFN-I regulators. In some cell types, IFN-Is induce the expression of AhR; as described by Yamada’s group, through a negative feedback mechanism, AhR inhibits IFN-Is response [77]. This point is of particular interest, as IFN-Is operate on two sides: (1) they induce IDO-1, which catalyzes the transformation of Trp in the immune-suppressive Kyn, which binds and activates AhR; and (2) they also induce the expression of AhR, which as anticipated, limits IFN-Is action via negative feedback. Thus, we can hypothesize that AhR could facilitate COVID-19 infection not only through Kyn-driven immune suppression, but also through the mitigation of IFN-Is response. 

### 6.2. NF-κB

AHR activity is negatively regulated by the TCDD-inducible poly-ADP-ribose polymerase (TIPARP), an enzyme that is induced after the binding between AhR and TCDD. Therefore, the *TIPARP* gene belongs to the AhR gene battery [78]. TiPARP is a mono-ADP-ribosyl transferase that transcriptionally inhibits AhR through the ribosylation of its core histones [79] (Figure 2). TIPARP catalyzes the ADP-ribosylation of other targets, including TANK- Binding Kinase (TBK1). This serine/threonine protein kinase has a key role in innate immunity, in particular during viral infections, as it coordinates the activation state of IRF3 (Interferon Regulatory Factor 3) and NF-κB. As this pathway is involved in IFN-I production and exocytosis, its inhibition by TIPARP-mediated ADP-ribosylation could indirectly link AhR to IFN-I inhibition in viral infections [77]. As anticipated, low levels of IFN-I have been reported in viral infections and, in particular, in COVID-19-affected patients [75]. A low capacity to induce IFN-I response is reported not only for SARS-CoV-2, but, more generally, for coronaviruses infections. These viruses have a set of proteins that can interfere with IFN-I production and functioning in target cells. Successful viral infection usually leads to the repression of IFN-Is, thus limiting the setting of the innate immunity. The inhibition of TBK1 mediated by TiPARP is not the only mechanism that can link AhR activation with IFN-Is inhibition. As anticipated, in 2020, Sa Ribero’s group [75] described that in SARS-CoV-2-infected cells, the RNA coronavirus interferes in IFNs production and signaling through different mechanisms that can target a) in infected cells: the intracellular sensing of the virus, the activation of cytoplasmic *IFNs* gene regulators, the induction of *IFN-I* (α/β) genes and their expression; and b) in surrounding cells, the IFN-Is binding to the interferon α and β Receptor (IFNAR) and the downstream pathway, which culminates in the induction and expression of *ISGs*. Some of these steps could potentially intersect with the AhR pathway: for example, after IFNAR activation, the downstream Jak/STAT pathway can recruit AhR [80]. In 2016, long before the COVID-19 outbreak, Rothhammer’s group described the involvement of AhR in the anti-inflammatory effect of IFN-Is in experimental models of CNS autoimmunity and in patients suffering from multiple sclerosis (MS). Here, it also described that AhR instigates the inhibition of NF-κB by inducing its inhibitor, SOCS2. AhR’s ability to modulate NF-κB during viral infections has been described in a paper by Giovannoni’s group [81]. Here, they showed that the activation of AhR from the Flavivirus Zika (ZIKV) negatively affects the production of IFN-I and the promyelocytic leukemia protein (PML), which drives the intrinsic immunity to ZIKV. In particular, AHR targets NF-κB, thus limiting its crosstalk with IFN-Is and PML, whose expression and antiviral functions are seriously affected. It has long been known that NF-κB is involved in the expression of TNFα and IL-6 induced by the Spike protein of SARS coronaviruses [82] in murine macrophages; in particular, according to Liao, the nucleocapsid S protein activates NF-κB [83]. This transcription factor is increasingly gaining a central role in the so-called COVID-19 “cytokine storm”, a cascade of inflammatory events that culminates in the systemic release of proinflammatory cytokines [84,85,86].

As described by a plethora of reports, AhR interacts with NF-κB and its family members RelA and RelB in various contexts, including the inflammatory context [62,87]. As evidenced by Poppe, in human CoVs infections, the NF-κB pathway can be prompted or suppressed. This complex strategy, which targets the host chromatin, is commonly present in RNA virus infections and is aimed at optimizing viral replication through the reprogramming of large sets of genes [88]. On this basis, it is possible to hypothesize that AhR and some key factors in the inflammatory response could be involved in this reprogramming. This hypothesis has been confirmed in a recent paper by Giovannoni’s group [89]. Here, considering previous studies, they used human and mouse cells infected with α and β Coronavirus. Here, they showed AHR activation by the increased expression of its cellular targets, including *CYP1A1* and *CYP1B1.* They also highlighted the rise in the expression of *AHR* and other genes linked to its pathway in mouse bone marrow-derived macrophages infected with a M(murine)-CoV β coronavirus and evidenced that SARS-CoV-2 replication is halted by the antagonists-mediated inhibition of AHR. These data were confirmed in cells from infected patients. The authors also detected an increase in *IDO-1* and *AHR* gene expression in cells from patients with medium/high gravity pathology, thus linking Kyn production to AhR activation and IDO-1-dependent tolerogenesis. More importantly, they evidenced a positive correlation between *AHR* gene expression and the viral load of infected cells. They showed that AHR regulates the transcription of a panel of genes linked to the inflammatory response, including NF-κB. In particular, they suggested that the activation of the AhR pathway limits the NF-κB-mediated immune strategy. Therefore, we can hypothesize that CoVs-mediated infections are facilitated from an inadequate immune response, due in part to the AhR-negative modulation of both IFN-I and NF-κB. This complex strategy is commonly present in RNA virus infections and is aimed at optimizing viral replication.

However, it must be considered that the progression of COVID-19 is rapid. The insufficient response by IFN-I allows for the evasion of the innate immune response for seven to ten days and for an increase in the viral load. Thus, monocytes accumulate in respiratory parenchyma and an increasingly intense and widespread inflammation develops, i.e., the so-called “cytokine storm”. According to Pallotta and Orabona [57,58], as anticipated, an increase in IL-6 drives IDO-1 to proteasomal SOCS-3-induced degradation; consequently, Kyn levels and Kyn-induced immune suppression decrease. In this IL-6-induced inflammatory environment, NF-κB is strongly induced by inflammatory mediators and plays an inflammatory role [82]. These issues encourage us to hypothesize a bi-modal interaction between AhR and NF-ĸB: a first phase, which allows for SARS-CoV-2 entry and replication, where AhR activates ACE-2 and has an inhibitory action on IFN-I. In this framework, the pp60src activation of IDO-1 favors the establishment of the IDO-1–Kyn–AhR circuit that prolongs the activation of AhR, whereas NF-κB plays an anti-inflammatory and immunosuppressive role. The protracted activation of AhR originates a proinflammatory loop, which sustains endogenous IL-6 production (Figure 1). The increase in IL-6 gradually involves other additional inflammatory pathways including the one sustained by NF-κB, whose proinflammatory influence gradually rises, playing a substantial role in the “cytokine storm”.

## 7. AhR Participates in Immune System Modulation in a Ligand-Dependent Modality

AhR regulates the adaptive immune response through the modulation of T cells differentiation. It has been demonstrated that AhR activation induces a shift in the balance between Treg/Th17 cells differentiation in a ligand-dependent modality. It has been shown that TCDD has an immunosuppressive effect, as the complex AhR/TCDD potentiates Treg differentiation; conversely, the binding between AhR and FICZ increases inflammation, as it endows Th17 generation [90,91]. Interestingly, Duarte from the Stockinger laboratory demonstrated that in an in vitro model of autoimmune encephalomyelitis, which is characterized by a high level of inflammation, both TCDD and FICZ are able to increase Th17 generation. A possible explanation could be that in in vivo models, P450 cytochrome-induced metabolic clearance is more efficient in removing FICZ, the effect of which is consequently shorter than TCDD [92]. This result evidences the importance of contextualizing experimental results, as metabolic processes can introduce significant variations in preliminary data obtained in cellular models. In the case of COVID-19, the shift in the Trp/Kyn ratio must also be considered, which in inflammation and cancer has been mainly ascribed to the upregulation of IDO-1 [93].

As a result of the constant subtraction of Trp from the extracellular milieu, which is paralleled by the increase in Kyn concentration, immune cell functionality declines and drives T cells toward apoptosis. Here, this metabolite triggers the apoptosis of T helper type 1 (Th1) cells, while favoring T helper type 2 (Th2) cell proliferation [94] (Figure 2). The /Kyn pathway has been described not only in cancer, but also in a discrete number of milieus, where it can be stimulated or inhibited. Depending on the cell context, it can originate products that interact with oxidative metabolism and the nervous and immune systems [95]. In COVID-19, the massive production of inflammatory cytokines drives the activation of IDO-1 and TDO; thus, it is possible to hypothesize that the production of Kyn could sustain the activation of AhR, which as we hypothesized, endows the endogenous production of IL-6 and the maintenance of the inflammatory state. This hypothesis is sustained by an observational study published by Thomas’ group [96]. Here, they checked serum metabolites of COVID-19-infected patients. They highlighted a relationship between patients’ inflammatory state, expressed as serum IL-6 and Protein C concentration, and as: (1) alterations in the amount of Trp transformation into Kyn; (2) the derangement of nitrogen metabolism, which is the expression of kidney function; (3) the alteration of the concentration of glucose and free fatty acids, which is representative of metabolic derangement. They reported an inverse proportionality between serum Trp and IL-6 concentrations. Here, the higher IL-6 levels, the lower were Trp, serotonin, and indole pyruvate levels. Elevated concentrations of Kyn, and kynurenic, picolinic, and nicotinic acids were indicative of the hyperactivation of the Trp processing pathway. Again, we remember that the serum Trp/Kyn ratio is representative of IDO-1’s enzymatic activity. This suggests that, in parallel with the increase in cytokines-mediated inflammation, which in symptomatic COVID-19, drives the so-called “cytokine storm”, there is a concomitant increment of an IDO-1-mediated transformation of the essential amino acid Trp in Kyn and derived metabolites. Considering these data, it could be interesting to know if in Thomas’ study, the activation of AhR is proportional to the increase in serum IL-6 and Kyn concentration and to follow the time course of their possible variation.

Considering that the transformation of Trp in Kyn is mainly catalyzed by IDO-1 and that Kyn is a well-known activating ligand for AhR, one could easily assume that in SARS-CoV-2 infection, the activation of AhR depends on IDO-1 enzymatic activity, through Kyn generation. This hypothesis has been questioned in 2020 by Grunewald’s group [97], as they also demonstrated AhR activation in murine IDO-1^-/-^ macrophages infected with Mouse Hepatitis Coronavirus (MHV). They concluded that in MVH infections, AhR activation is not IDO-1-dependent, thus excluding that Kyn could be the activating ligand of AhR. Consequently, similarly to Turski [71], they hypothesized the presence of (an) unknown activating ligand(s) that drive(s) the participation of AhR to an MHV-induced inflammatory state. In Grunewald’s study, the inactivation of AhR by TiPARP-mediated ADP-ribosylation decreases the level of inflammation, thus giving a clear indication about the proinflammatory role of AhR in MVH infection. In our opinion, in this case, some inflammatory mediators, i.e., IL-6 [60,61,62], could be AhR activators, according to the above-mentioned study by Stobbe-Maicherski’s group [60]. However, in a recent research highlight from Giovannoni and Quintana [98], the authors, considering recent data, underline: (1) the upregulation of TDO/IDO in viral infection; (2) Kyn-induced AhR activation; (3) its limiting role in the immune response mediated by IFN-I and NF-κB in viral infections comprising Zika virus, influenza A virus and, with high probability, SARS-CoV-2 virus. In this last case, they also support Liu’s hypothesis [52] that states that activated AhR drives the increase in mucin production in lung cells, thus proposing this transcription factor as a possible therapeutic target for relieving respiratory symptoms in COVID-19-affected patients. Consistently, the altered Trp/Kyn is a distinguishing feature in COVID-19-positive patients [99]. Thus, a question arises concerning the role of Kyn in this intricate framework. The transformation of Trp in Kyn can be catalyzed by TDO or IDO-1, but in the immune system, the expression of *IDO-1* is prevalent and can be induced by viral infections and IFN-γ. In the case of COVID-19, the data from Grunewald [97] and Turski [71] can lead us to consider some inflammatory mediators as potential intracellular inductors of IDO-1, the activation of which is the logical explanation for Kyn overproduction. Therefore, in the COVID-19 inflammatory environment, it is possible to imagine that AhR engagement is achieved by Kyn activation and/or by a possible IL-6-dependent STAT-3 modulation of *AhR* gene expression (Figure 1) (61). This last hypothesis, although suggestive, still needs supporting data, especially in the context of virus-derived inflammation. STAT-3 also binds to the *IDO* promoter and activates *IDO-1* expression (Figure 1); thus, as we propose, AhR activation is probably a result shared by different pathways. It is widely accepted that the balance of Trp/Kyn concentrations impacts immune system regulation and is a measure of inflammation level [100]. In particular, as anticipated, in Chen’s report [63], a distinguishing feature of severe and moderate COVID-19 infection is the high inflammatory state, which is the result of a sort of self-feeding escalation where inflammation and immunosuppression are contemporary phenomena that are mutually reinforcing. In light of the data presented thus far, the AhR pathway could be a likely link between these two aspects.

## 8. Concluding Remarks and Future Perspectives

In this review, we describe some experimental evidence and clinical data suggesting AhR’s involvement in COVID-19’s hyper-inflammation and in the imbalance in host antiviral defense. We built this working hypothesis considering that AhR activation depends not only on xenobiotics but also on endogenous molecules, including inflammatory cytokines and chemokines. From this perspective, we analyzed the possible participation of AhR in the biochemical inflammatory signature of this pathology and in its unbalanced immune response, considering its relationships with some inflammatory key players. In particular, we evaluated its interaction with IDO-1-derived Kyn and the IL-6 pathway. We also described the possible interference of AhR in the immune response through the modulation of IFN-I and NF-κB, the alteration of DC cells’ immunogenicity and the alteration of the Tregs/Th17 cell ratio in a ligand-dependent modality.

The lack of selectivity toward ligand binding and its ligand-independent activation could allow for the participation of AhR in different inflammatory settings that develop in different body districts involved in COVID-19 infection. This last cue should lead to caution when analyzing the combined effects of the possible molecular players of COVID-19. Moreover, AhR can be activated by a multitude of exogenous ligands, both of environmental and/or anthropic origin, which might significantly interfere with the mechanisms described thus far [101]. Pollutants are widespread in all environmental matrices, through which they can interact with the human body. Consequently, a large number of them can potentially interact with AhR and contribute to the worsening of COVID-19-related symptoms and risk factors [102]. This issue has been recently hypothesized in order to explain the high incidence of COVID-19 cases in highly polluted areas of Northern Italy during the first phase of this pandemic [103]. Considering the relevance of this topic and its impact on global health and the global socio-economic situation, further studies are urgently needed in order to clarify the potential role of AhR in the onset and progression of COVID-19 and, in this framework, to evaluate the outcome of possible interactions with environmental pollutants.

## Abbreviations

ABCG2	ATP-binding cassette super-family G member 2
ACE-2	Angiotensin- Converting Enzyme 2
AhR	Aryl hydrocarbon Receptor
AhRR	Aryl hydrocarbon Receptor Repressor
AIP	Immunophilin-like Ah Receptor-interacting protein
ALDH3A1	Aldehyde dehydrogenase family 3, subfamily 1
AP1	Activator protein 1
AR	Androgen Receptor
ARNT	Aryl hydrocarbon Nuclear Translocator
bHLH	basic Helix-Loop-Helix
Blimp1	B lymphocyte-induced maturation protein-1
B°AT1	Broad neutral Amino acid Transporter 1
CD36	Cluster of differentiation 36
CD38	Cluster of differentiation 38
CDC	Center for Disease Control and Prevention
c-Kit	tyrosine-protein kinase KIT
CNS	Central Nervous System
COVID-19	Coronavirus-Induced Disease-2019
COX2	Cyclooxygenase-2
CpG	C phosphate G
CSE	Cigarette Smoke Extract
CTS	Cathepsin
CXCL5	C-X-C motif chemokine 5
CYP1A1	Cytochrome P450 Family 1 Subfamily A Member 1
CYP1A2	Cytochrome P450 Family 1 Subfamily A Member 2
CYP1B1	Cytochrome P450 Family 1 Subfamily B Member 1
CYP19A1	Cytochrome P450 Family 19
DC	Dendritic Cell
DHT	Dihydrotestosterone
DRE	Dioxin Responsive Elements
FICZ	6-formylindolo [3,2-b] carbazole
Gstα1	Glutathione S-transferase, alpha 1
GM-CSF	Granulocyte-Macrophage Colony-Stimulating Factor
HAHs	Halogenated Aromatic Hydrocarbons
HepG2	Hepatoma G2
HIF1-α	Hypoxia Inducible Factor-1α
HSP90	Heat Shock Protein 90
IDO-1	Indoleamine-2,3-Dioxygenase
IFN	Interferon
IFNAR	Interferon alpha and beta Receptor
IFN-I	Type I Interferon
IDO-1	Indolamine 2,3 dioxygenase
IL	Interleukin
IL-1β	Interleukin 1β
IL-1R	Interleukin 1 receptor
IL-6ST	Interleukin-6 receptor subunit beta precursor
IRF	IFN Regulatory Factor
ISGs	Interferon-Stimulated Genes
ISGF3	IFN-stimulated growth factor 3
ISREs	Interferon-Stimulated Response Elements
Kyn	Kynurenine
LAT-1	L-type amino acid antiporter 1
LPS	Lipopolysaccharide
MACROD1	Macrodomain Containing Proteins 1
MAPK	Mitogen-Activated Protein Kinase
MDA-5	Melanoma Differentiation-Associated Gene 5
MHC II	Major Histocompatibility Complex of II type
MMP	Matrix Metalloproteinase
mRNA	Messenger Ribonucleic Acid
MS	Multiple Sclerosis
MHV	Mouse Hepatitis (corona) Virus
NF-kB	Nuclear Factor kappa-light-chain-enhancer of activated B cells
NLS	Nuclear Localization Signal
NRF2	Nuclear factor erythroid 2-related factor 2
OMP	Omeprazole
p21Cip	cyclin-dependent kinase inhibitor 1
p23	Proteolytically Resistant 23-kDa protein
p27^Kip1^	Cyclin-dependent kinase inhibitor 1B
p40phox	Neutrophil cytosol factor 4
PAHs	Polycyclic Aromatic Hydrocarbons
PARP	Poly ADP Ribose Polymerase
PDCs	Plasmacytoid Dendritic Cells
PML	Promyelocytic Leukemia Protein
PP60src	Proto-oncogene tyrosine-protein kinase 60 (Sarcoma)
PSA	Prostate-specific antigen
RelA	V-Rel Reticuloendotheliosis Viral Oncogene Homolog A
RelB	V-Rel Reticuloendotheliosis Viral Oncogene Homolog B
RIG-1	Retinoic Acid-Inducible Gene I
RLSs	Retinoic acid-inducible gene I (RIG-1)-like receptors
SARS-COV-2	Severe Acute Respiratory Syndrome Coronavirus-2
SOCS	Suppressor of cytokine signaling
STAT	Signal Transducer and Activator of Transcription
TBK1	TANK Binding Kinase
TCDD	2,3,7,8-Tetrachlorodibenzo-p-Dioxin
TDO2	Tryptophan 2,3-dioxygenase
TGF-β	Transforming growth factor beta
Th17	T helper 17 cells
TiPARP	TCDD-inducible Poly ADP-Ribose Polymerase
TMPRSS2	Transmembrane protease, serine 2
TNF-α	Tumor Necrosis Factor-α
T_R_1	Type 1 Regulatory T Cells
Treg	regulatory T cells
Trp	Tryptophan
Ugt1a6	UDP glucuronosyltransferase family 1 member A6
WHO	World Health Organization
XAP2	Hepatitis B virus X-Associated Protein 2
XREs	Xenobiotic Response Elements
ZIKV	Flavivirus Zika
